# Insights into the Age Dependency of Compositional MR Biomarkers Quantifying the Health Status of Cartilage in Metacarpophalangeal Joints

**DOI:** 10.3390/diagnostics13101746

**Published:** 2023-05-16

**Authors:** Miriam Frenken, Karl Ludger Radke, Emilia Louisa Ernestine Schäfer, Birte Valentin, Lena Marie Wilms, Daniel Benjamin Abrar, Sven Nebelung, Petros Martirosian, Hans-Jörg Wittsack, Anja Müller-Lutz

**Affiliations:** 1 Department of Diagnostic and Interventional Radiology, Medical Faculty, University Hospital of Dusseldorf, D-40225 Dusseldorf, Germany; miriam.frenken@med.uni-duesseldorf.de (M.F.);; 2 Department of Orthopedics and Trauma Surgery, Medical Faculty, University Hospital of Dusseldorf, D-40225 Dusseldorf, Germany; 3 Department of Diagnostic and Interventional Radiology, University Hospital Aachen, D-52074 Aachen, Germany; 4 Department of Diagnostic and Interventional Radiology, Medical Faculty, University Hospital of Tübingen, D-72076 Tübingen, Germany

**Keywords:** MRI, cartilage, metacarpophalangeal joints, relaxation times, T1ρ, age, musculoskeletal imaging

## Abstract

(1) Background: We aim to investigate age-related changes in cartilage structure and composition in the metacarpophalangeal (MCP) joints using magnetic resonance (MR) biomarkers. (2) Methods: The cartilage tissue of 90 MCP joints from 30 volunteers without any signs of destruction or inflammation was examined using T1, T2, and T1ρ compositional MR imaging techniques on a 3 Tesla clinical scanner and correlated with age. (3) Results: The T1ρ and T2 relaxation times showed a significant correlation with age (T1ρ: Kendall-τ-b = 0.3, *p* < 0.001; T2: Kendall-τ-b = 0.2, *p* = 0.01). No significant correlation was observed for T1 as a function of age (T1: Kendall-τ-b = 0.12, *p* = 0.13). (4) Conclusions: Our data show an increase in T1ρ and T2 relaxation times with age. We hypothesize that this increase is due to age-related changes in cartilage structure and composition. In future examinations of cartilage using compositional MRI, especially T1ρ and T2 techniques, e.g., in patients with osteoarthritis or rheumatoid arthritis, the age of the patients should be taken into account.

## 1. Introduction

Magnetic resonance imaging (MRI) is suitable for assessing cartilage quality and can therefore be used to evaluate cartilage disorders [1]. In particular, diseases such as rheumatoid arthritis (RA) and osteoarthritis (OA) can be associated with cartilage degeneration in the metacarpophalangeal (MCP) joints [2,3]. However, changes in cartilage quality may also occur during the normal aging process [4,5]. Therefore, it is useful to know whether and how age-related cartilage changes are reflected in magnetic resonance biomarkers in MCP joints. This could allow better differentiation between age-related and disease-related cartilage changes in the MCP joints.

Various magnetic resonance techniques allow the quantification of cartilage composition and structure. These methods include the determination of the longitudinal relaxation time T1, the relaxation time in the rotating frame T1ρ and the transverse relaxation time T2, chemical exchange saturation transfer (CEST) imaging, and sodium imaging [6,7,8,9,10]. Potential age-related changes in proteoglycan content and cartilage structure could affect these parameters, as was already shown for T2 and T1ρ [11,12].

The longitudinal relaxation time T1 could be sensitive to the cartilage structure, as it represents the proton mobility, which, in turn, depends on the shape of the extracellular matrix.

Change in the components in articular cartilage can also be detected with the relaxation time in the rotating frame T1ρ. With T1ρ mapping, changes in proteoglycans can be identified, as T1ρ relaxation times are negatively related to the proteoglycan content, as has already been shown in various studies [13,14,15]. In addition, T1ρ was shown to correlate moderately with water content [13], and in OA patients, T1ρ values were higher compared to healthy volunteers [16]. 

Quantitative T2 mapping shows wide utility in the characterization of cartilage [17]. The transverse relaxation time T2 is dependent on the water content and its interaction with the extracellular matrix [18]. Magnetic resonance experiments quantifying the T2 relaxation time during the aging process in articular cartilage in the knee have been performed by Mosher et al. [12]. They found an increase in T2 values in articular knee cartilage with increasing age after the age of 45 in a cohort of asymptomatic women [12]. They proposed that this increase is due to a reduction in the anisotropy of the collagen fibers or an increase in the mobility of the cartilage water. In addition, they suggested that the aging of cartilage collagen begins near the joint surface and progresses to the deeper cartilage layers with age.

In addition, there are various age-related changes in the cells and the extracellular matrix of articular cartilage, such as proteolysis, reduction in cell density, or cellular senescence with abnormal secretion profiles [19], of which, to our knowledge, no causality to the T2 elevation has been proven so far, but which is conceivable due to the presumed increase in extra- and intracellular water content.

In line with the results for knee articular cartilage by Mosher et al. [12,20], we can also expect elevated T2 values for finger cartilage.

CEST imaging has already been shown to be related to the content of glycosaminoglycans [21]. However, the gagCEST effect in articular cartilage at a field strength of 3 Tesla is very small [22]. In addition, CEST imaging is highly susceptible to artifacts due to field inhomogeneities [23], making this method difficult to apply in MCP joints.

Sodium imaging of cartilage is based on the idea that the GAG side chains of proteoglycans create a negative fixed charge density (FCD) that attracts positively charged sodium ions [10]. This makes sodium imaging very attractive for the assessment of degenerative processes in articular cartilage since degeneration results in a decrease in sodium ions in the tissue [14]. However, due to the low intrinsic resolution, long imaging times, and special hardware requirements [24,25], this method is again difficult to perform in MCP joints. In addition to MRI imaging, the MCP joints can also be examined using ultrasound. Ultrasonography appears to increase the accuracy of assessment of the MCP joints in RA compared to conventional radiography [26]. Joint changes such as osteophytes and erosions can occasionally also be found in healthy subjects [27]. 

In comparison, both morphological MRI imaging and ultrasound are important because both can detect early disease stages in RA [28]. A direct comparison between ultrasound and compositional MRI biomarkers is not known to us.

Our study aims to evaluate if the compositional magnetic resonance imaging techniques T1, T1ρ, and T2 can detect age-related changes in articular cartilage in MCP joints. Furthermore, compositional magnetic resonance measurements may indicate specific structural changes in the MCP joints, such as changes in water content or composition of the extracellular matrix. Moreover, a possible age-related extent of T1, T1ρ, and T2 changes should be compared with expected disease-related cartilage changes, such as OA or RA.

## 2. Materials and Methods

### 2.1. Study Population

This study was designed and conducted as a prospective compositional magnetic resonance biomarker in vivo study of a general longitudinal cohort of adult volunteers. The subjects were recruited from the study leader’s extended work environment and circle of friends. Our primary objective was to investigate the effectiveness of magnetic resonance imaging biomarkers in assessing the health status of the cartilage in the metacarpophalangeal joints (MCP) of digits 2 to 4 in 30 healthy subjects and validate these biomarkers with respect to age-related changes. This study was approved by the local ethics committee (Medical Faculty, University of Düsseldorf, Germany, study number 2021-1363), and written informed consent was obtained from all participants.

Prior to enrollment in the study, all subjects were questioned about pre-existing conditions and surgeries in the examination region and excluded if necessary. Based on the morphological magnetic resonance images, they were carefully controlled to ensure that they showed no signs of degenerative changes, particularly with regard to cartilage or joints.

### 2.2. Hardware and Sequence Protocol

**Figure 1:**
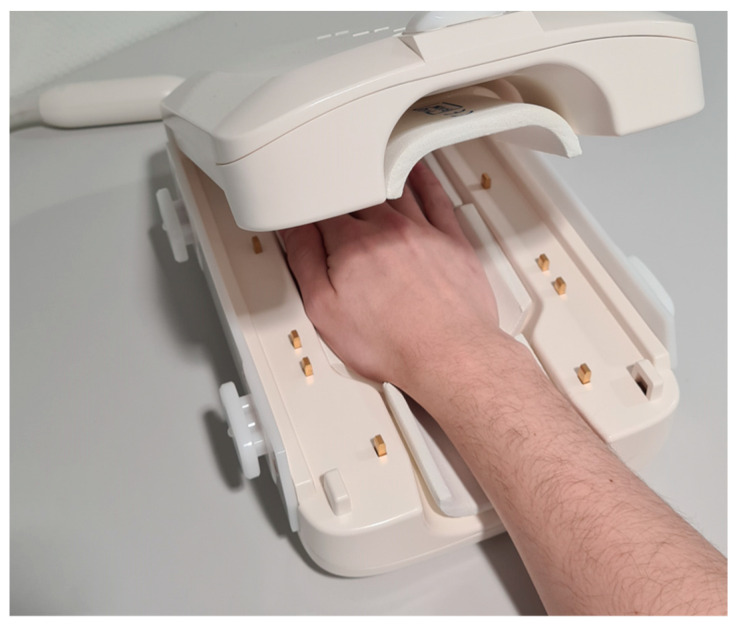
Positioning of the right hand of a volunteer in the ‘superman position’.

All volunteers were examined in a prone position with the right hand extended over the head (‘superman position’) using a clinical 3 Tesla magnetic resonance scanner (MAGNETOM Prisma, Siemens Healthcare, Erlangen, Germany) (see Figure 1). Signal reception was performed with a dedicated 16-channel hand/wrist coil for high-resolution hand and wrist imaging (Hand/Wrist 16, Siemens Healthcare, Erlangen, Germany).

**Figure 2:**
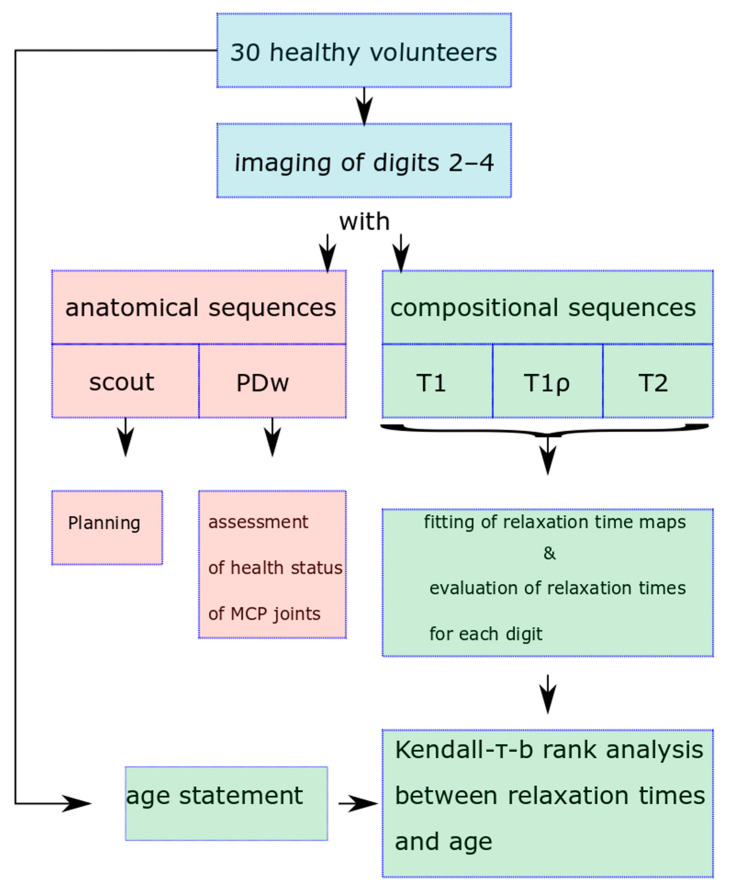
Visualization of study design.

Figure 2 displays a flowchart summarizing the data acquisition and post-processing steps of our study.

After performing a scout, a proton-density-weighted sequence was acquired to assess the overall health of the MCP joint within a field of view of 140 × 140 × 44 mm^3^ in all study participants. In addition to the proton density sequence, compositional magnetic resonance sequences with an in-plane resolution of 0.5 × 0.5 mm and a slice thickness of 4.5 mm were acquired. These compositional sequences include a T1, T1ρ, and T2 sequence. These sequences were acquired in a single central slice planned on the scout, and the results of the proton-density-weighted sequence provide detailed information about the MCP joint’s composition. Details on the sequence parameters are provided in Table 1.

To obtain T1 mapping, an inversion recovering protocol including five different sequences with an inversion recovery preparation and a repetition time TR of 5190 ms was acquired. These five different sequences were acquired with five different inversion times (TI) ranging between 25 and 2000 ms, as detailed in Table 1. T1 mapping provides information on the longitudinal relaxation time of the joint tissue, which can be used to assess tissue composition and detect early degenerative changes.

For T2 relaxation time measurements, ten different T2 preparation pulses ranging from 0 ms to 90 ms with a step size of 10 ms were used. For T1ρ acquisitions, we used ten different spin-lock times (TSL), which also range from 0 ms to 90 ms with a step size of 10 ms. T1ρ and T2 sequences are highly sensitive to changes in the joint’s proteoglycan content and fluid accumulation, respectively. By utilizing multiple compositional magnetic resonance sequences, including T1, T1ρ, and T2, this study was able to provide a comprehensive evaluation of the MCP joint’s composition, enabling the detection of even subtle changes in the joint tissue.

### 2.3. Data Analysis

Using ITK-SNAP software (v3.8.0, Cognitica, Philadelphia, PA, USA, www.itksnap.org (accessed on: 29 April 2021)) [29], regions of interest (ROI) were drawn separately for MCP joints of the index, middle, and ring finger (MCP2, MCP3, and MCP4) by two radiologists (1. MF, six years; 2. BV, four years of musculoskeletal radiology). The first radiologist drew the ROIs twice (4 weeks after the initial assessment) to assess intrareader reliability.

ROIs were transferred to the externally calculated relaxation maps. To produce relaxation maps, the fitting routines were implemented in Python (v3.9, Python Software Foundation, Wilmington, DE, USA), and non-linear least-square fits were applied voxel-wise to the exponential decay curves. To validate fit quality, coefficient of determination (R^2^) statistics adjusted to the degrees of freedom were calculated, and only voxels with R^2^-values ≥ 0.75 were included in the subsequent analysis.

For the calculation of T1 maps, the signal equation

(1) $$S\left(TI\right)=S_{0}(1-2\exp\left(-\frac{TI}{T1}\right))$$

was used. According to Rauscher et al. [30], we determined T1ρ and T2 by fitting signal intensities with the following equations [30,31]:

(2) $$S\left(TSL\right)=S_{0}\sin\left(\alpha \right)\frac{\exp\left(-\frac{TSL}{T1\rho }\right)\left(1-\exp\left(-\frac{TR-TSL}{T1}\right)\right)\exp\left(-\frac{TE}{T2^{*}}\right)}{1-\exp\left(-\frac{TSL}{T1\rho }\right)\exp\left(-\frac{TR-TSL}{T1}\right)\;\cos\left(\alpha \right)}+const.$$

(3) $$S\left(T2_{prep}\right)=S_{0}\sin\left(\alpha \right)\frac{\exp\left(-\frac{T2prep}{T2}\right)\left(1-\exp\left(-\frac{\mathrm{TR}-T2\mathrm{prep}}{T1}\right)\right)\exp\left(-\frac{TE}{T2^{*}}\right)}{1-\exp\left(-\frac{T2prep}{T2}\right)\exp\left(-\frac{\mathrm{TR}-T2\mathrm{prep}}{T1}\right)\cos\left(\alpha \right)}+const.$$

For both calculations, we used TR = 5000 ms, α = 15°, and TE = 2.59 ms, as shown in Table 1. Furthermore, we corrected the fitting with respect to T1, as indicated in the formula, using T1 = 900 ms.

### 2.4. Statistical Analysis

The statistical analyses were conducted by KLR using R software (v4.1.3, R Foundation for Statistical Computing). The Kendall-τ-b rank correlation analysis was performed to investigate the obtained mean T1, T1ρ, and T2 times as a function of age. Based on Cohen et al., the tau effect size was classified as weak (0.1–0.3), moderate (0.3–0.5), and strong (>0.5) [32]. 

In cases where a significant correlation was detected, we determined the dependence between the corresponding relaxation time and age in the form of a straight-line equation. With this foundation, we were then able to estimate the expected percentage of change in these relaxation times between 20 and 30 years, 20 and 40 years, 20 and 50 years, 20 and 60 years, 30 and 40 years, 30 and 50 years, 30 and 60 years, 40 and 50 years, 40 and 60 years, and 50 and 60 years. 

To measure relative reliability, we calculated the intraclass correlation coefficient (ICC) and adopted the classification according to Koo et al. ICC values were classified as poor (ICC < 0.5), moderate (0.5 <= ICC < 0.75), good (0.75 <= ICC < 0.9), and excellent (ICC >= 0.9) [33]. For the purpose of determining inter-rater reliability, we used ICC(2, 1), whereas ICC(3, 1) was applied to assess intra-rater reliability [34]. 

All data are presented as median (min–max). To ensure the validity of our findings, we considered *p*-values ≤ 0.05 as the threshold for statistical significance.

## 3. Results

In our study, 30 healthy volunteers were included (mean age: 44 ± 14 years, range: 20–68 years, 17 females, 13 males). Of these, 29/30 were Caucasian, 0/30 were Afro-American, and 1/30 were of Asian descent.

Data acquisition was successfully performed in the whole study cohort. Table 2 provides the descriptive statistic of magnetic resonance parameters for all subjects and MCP joints. With 918.8 (766.7–1090.2) ms, the longitudinal relaxation time was much higher compared to the relaxation times T1rho 19.6 (15.0–32.6) ms and T2 15.6 (9.3–25.7) ms.

**Figure 3:**
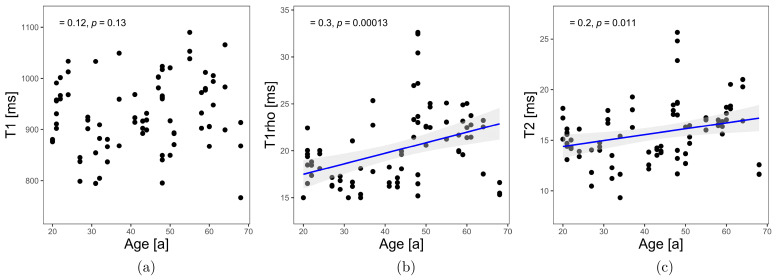
Correlation analysis between T1 (**a**), T1ρ (**b**), and T2 (**c**) as function of age in MCP joints.

For the relaxation times T1, our study results show no dependence on age (T1: Kendall-τ-b = 0.12, *p* = 0.13). Significant positive correlations were shown between relaxation times T2, and T1ρ and age (T2: Kendall-τ-b = 0.20, *p* = 0.01, weak positive correlation with age; T1ρ: Kendall-τ-b = 0.30, *p* < 0.001, moderate positive correlation with age) (see Figure 3). The straight-line equations for the relaxation times T1ρ and T2 result in

(4) $$T1\rho \left(age\right)=\left(0.112\;\pm 0.029\right)\left[\frac{ms}{years}\right]*age\left[years\right]+\left(15.249\;\pm 1.285\right)\left[ms\right]$$

and

(5) $$T2\left(age\right)=\left(0.058\;\pm 0.022\right)\left[\frac{ms}{years}\right]*age\left[years\right]+\left(13.230\;\pm 0.988\right)\left[ms\right].$$

Therefore, a higher increase in relaxation times with age is observed for T1rho compared to the transverse relaxation time T2. The expected percentage increase in T1ρ and T2 values with age is summarized in Table 3. Over a period of 40 years, for example, T1ρ times are expected to increase by 25.6%, whereas transverse relaxation times T2 increase by only 16.1% over this period. 

**Figure 4:**
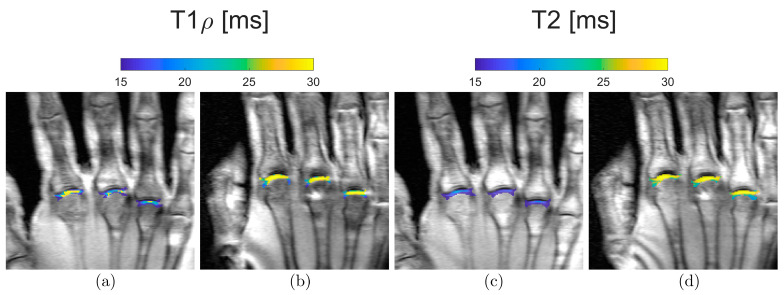
Representative quantitative mapping of relaxation times T1ρ (**a**,**b**) and T2 (**c**,**d**) of a 21-year-old (**a**,**c**) and a 55-year-old subject (**b**,**d**). Blue, i.e., low T1ρ and T2 values, can be seen in the MCP joints of the younger volunteer. In contrast, yellow, i.e., high T1ρ and T2 values, predominate in the MCP joints of the older participant.

Weak and moderate changes can be visualized by quantitative mapping of T2 and T1ρ. A 21-year-old subject and a 55-year-old subject exemplify this (Figure 4). Higher relaxation times in the MCP joints can be observed for T1ρ and T2 relaxation times in the 55-year-old volunteer compared to the 21-year-old participant.

Excellent intra-rater reliability and moderate inter-rater reliability was obtained for T1ρ (intra-rater: ICC(3, 1) = 0.99 (95% CI = 0.99–0.99); inter-rater: ICC(2, 1) = 0.71 (95% CI = 0.48–0.83)). We found excellent intra-rater reliability and good inter-rater reliability for T2 (intra-rater: ICC(3, 1) = 0.98 (95% CI = 0.97–0.99); inter-rater: ICC(2, 1) = 0.75 (95% CI = 0.62–0.83)). We observed good intra-rater reliability but poor inter-rater reliability for T1 (intra-rater: ICC(3, 1) = 0.91 (95% CI = 0.86–0.94); inter-rater: ICC(2, 1) = 0.39 (95% CI = −0.19–0.56)).

## 4. Discussion

The main finding of this study is that age-related changes in cartilage structure and glycosaminoglycan content of cartilage can be assessed by compositional magnetic resonance imaging, even in small MCP-finger joints. Furthermore, our study results support the concept of decreasing glycosaminoglycan content with age, even in healthy volunteers in the cartilage of the MCP joints.

In this study, three different MR sequences were examined (T1, T1 ρ, and T2). Compared to other studies, the T2 values obtained in our study in the MCP joints were relatively low (range of 9.3 ms–25.7 ms). Higher T2 levels were predominantly observed in the articular cartilage of patients with joint pain, osteoarthritis (OA), or rheumatoid arthritis (RA), where higher T2 values were observed [35,36,37]. The higher T2 values observed in these studies could, on the one hand, be due to the fact that, unlike the present study, they involved patients with diseases or symptoms in the finger joints, such as pain, OA, or RA. Meng et al. reported that in the case of cartilage damage, higher T2 values could be expected in articular cartilage [38]. On the other hand, the magnetization-prepared T2 mapping sequence we used with T2 preparation could also explain the lower observed T2 values, as studies have already reported that sequences with T2 preparation result in lower T2 values compared to standard T2 mapping with multi-echo sequences [39,40]. The advantage of T2 mapping with magnetization preparation is the robustness of the method to B1 field inhomogeneities [39]. Since such field inhomogeneities are to be expected, especially in the small metacarpophalangeal joints, we decided to use this B1-insensitive sequence. Nevertheless, caution should be exercised when comparing transverse relaxation times T2 between different studies using different magnetic resonance sequences [41].

Transverse relaxation (T2) times are dependent on both hydration status [42,43] and the organization of collagen fibers [12]. In the present study, we observed a significant increase in T2 relaxation times as a function of age. Similarly, an increase in T2 relaxation times was also observed in the articular cartilage of the knee joint in patients with OA compared to healthy volunteers [18]. Additionally, Mosher et al. showed increasing T2 values in patellar cartilage with age in a cohort of thirty asymptomatic women who were between 22 and 86 years old [12]. Mosher et al. divided the cartilage into different anatomical regions and examined the T2 values in relation to the different areas [12]. In the cartilage center, they observed a 9.3% increase in T2 times between a cohort aged 18–30 years and a cohort aged 45–65 years, whereas the increase was greater at the cartilage surface (20.6%) [12]. We observed a 12.09% increase in T2 between 20 and 50 years of age in our study, placing it well within the range expected by the literature. Due to the limited resolution and the small size of the cartilage layer in the MCP joints, a separate measurement between the surface and the center of the cartilage is not possible at this stage.

Different studies investigated the transverse relaxation times of T2 and their dependencies in OA and RA. For example, Renner et al. showed that anticitrullinated-protein-antibodies-positive RA patients have approximately 39–55% higher T2 relaxation times in the metacarpal heads than anticitrullinated-protein-antibodies-negative patients [37]. In comparison, age-dependent changes in the T2 transverse relaxation times observed in our study are relatively small. Kretschmar et al. performed a study analyzing the cartilage of OA patients in more detail and showed that even before the appearance of a lesion, there is an approximately 10–12% increase in T2 values compared to the surrounding non-arthritis knee cartilage [36]. This increase in T2 relaxation time is in the order of magnitude that we expect to see in our study due to the aging process over about 30 years.

The second MR sequence we analyzed was T1ρ. This study showed a significant increase in T1ρ values with age. T1ρ imaging depends on the GAG concentration and, to a lesser degree, on the chemical exchange process between protons of GAG and water [14,44,45]. Therefore, it is possible to consider T1ρ as a surrogate parameter for GAG content. Keenan et al. and Wong et al. detected a negative correlation between GAG content and T1ρ values in the cartilage of the knee joint [11,46]. In addition, Keenan et al. observed a significant decrease in GAG content with age in all anatomical regions of patellar cartilage of human specimens [11]. In this respect, the results of our study are in line with the literature, and we hypothesize that our positive correlation between T1ρ and age is caused by a decrease in GAG content.

In the literature, T1ρ is frequently applied to the study of RA and OA [11,47,48,49,50]. The T1ρ values of these studies range from about 14 ms to 100 ms, where higher values are associated with reduced GAG content [11,47,48,49,50]. Wang et al. observed significantly higher T1ρ values in the knee of patients with advanced degeneration (whole-organ MR imaging score (WORMS) = 5–6) compared to patients with doubtful or minimal degeneration (WORMS = 0–1) [47]. Tsushima et al. performed a macroscopic grading of cartilage sample tissues and compared the T1ρ values for similar macroscopic grades between patients with OA and RA [48]. They could detect significantly higher T1ρ values for patients with RA compared to patients with OA, whereby the changes in the superficial layer were higher compared to the deep layer [48]. In RA patients, treatment by tumor necrosis factor alpha (TNF alpha) inhibition can be used. Ku et al. were able to detect treatment-associated cartilage changes by means of T1ρ imaging and classified the changes based on the ‘European League Against Rheumatism’ treatment response criteria [49]. Thereby, a good response caused a small reduction in T1ρ (about 2 ms), whereby non-responders showed a small increase in T1ρ (about 2 ms) [49]. In consideration of the present study and the studies in the literature, it must be concluded that T1ρ changes due to age are not negligible and are in a range, which is clinically relevant. 

Contrary to the transverse relaxation times T2 and the T1ρ relaxation times, we observed no significant changes in longitudinal relaxation times T1 in the MCP joints as a function of age. T1 and T2 mapping can measure different magnetic resonance relaxation mechanisms, so T1 values provide complementary information about macromolecular changes in cartilage [18]. In earlier studies, Buchbender et al. did not find any differences in T1 values in the MCP joint of the index finger between healthy volunteers and RA patients [51]. Although T1 imaging has been technically improved, for example, with higher resolution, in our study, it still does not appear to be sensitive enough in the small finger joints to detect age-related changes. To our knowledge, there are no other studies to date in which T1 mapping has been performed in the finger joints. In the greater shoulder joint, Cao et al. observed a significant correlation between T1 mapping and MRI-based degeneration grading [52]. However, his study also showed a higher correlation of T2 relaxation times than T1 times with MRI-based degeneration grading in the shoulder, suggesting that the T2 mapping value is most important for quantitative analysis of articular cartilage degeneration [52].

We would like to emphasize that in this study, we were able to determine the cartilage composition by quantitative relaxation time measurement in the very thin cartilage of the MCP joints. This is challenging as partial volume effects can occur in small cartilage structures. To be precise, there are two cartilage structures in the MCP joint, one of which can be assigned to the Os Metacarpi and the other to the Phalanx proximalis. Between these two cartilage structures, there is a small amount of physiological synovial fluid inside the joint capsule to allow smooth movement within the joint [53]. We used a dedicated hand/wrist coil for high-resolution magnetic resonance imaging to prevent partial volume effects. Our acquired in-plane high-resolution (0.5 × 0.5 mm^2^) sequence may not have been sufficient to completely separate the synovial fluid from the cartilage layer. Therefore, the relaxation times of the synovial fluid may have influenced our results. In the future, advanced techniques for partial volume correction, such as the driven equilibrium single-shot observation of T1 and T2 (ms) [54], might be investigated. However, much longer relaxation times are expected in synovial fluid compared to cartilage [55], so we can assume that the influence of synovial fluid on our measured relaxation times is very small.

In the literature, dGEMRIC (delayed gadolinium-enhanced magnetic resonance imaging of cartilage) is often used to quantify the GAG content of cartilage, and this technique has also been successfully applied in RA and OA patients as well as in patients with psoriatic arthritis [56,57]. Although this technique has great potential to quantify cartilage composition, we made a conscious decision not to use this technique in healthy subjects for ethical reasons to avoid the use of contrast agents.

Our study has several limitations: 1. We performed no measurements of reproducibility. In our study, all subjects were placed in the prone position with the hand held out above the head. Remaining in this position for the entire measurement time is strenuous for the volunteers, which is why we did not measure reproducibility. 2. We did not measure the biochemical imaging methods CEST and sodium MRI. There are various reasons for this: the CEST effect in articular cartilage is very low [22]. Due to the high field inhomogeneities to be expected, this technique is difficult to apply in MCP joints. For measurements with sodium imaging, we would have had to modify the measurement setup, as dedicated hardware is required for this purpose. In addition, sodium MRI acquisitions are usually relatively long [25], so the overall measurement protocol would become too long. However, it is conceivable to investigate the age dependence of sodium content using sodium MRI in MCP joints in a future study. 3. All measurements were performed in the right hand, independent of the handedness of the subjects. However, the handedness might affect the observed results, as Solovieva et al. showed a protective effect of moderate hand use in patients with OA [58]. In addition, handedness influences musculoskeletal structures such as knee joint cartilage [59]. 4. The cartilage layer in the MCP joints is very small (between 0.3 and 0.9 mm [60]); thus, partial volume artifacts might occur. Nevertheless, we were able to demonstrate age-dependent changes in the relaxation times T2 and T1ρ, indicating that the partial volume effect did not appear to have a significant impact on our results. 5. We did not perform histological validation of GAG content because we could not represent cartilage harvesting from healthy volunteers for ethical reasons. 6. This study might have been improved by an increased number of volunteers. However, the acquisition of volunteers is particularly difficult in older age groups since underlying diseases such as arthrosis or arthritis are often already present. 7. Because of the small cohort size, we did not consider sex-related differences in relaxation times. 8. It has now been proven that smoking has a negative effect on cartilage, and this can be reflected in increased T2 values [61]. We have not yet addressed this in the current study.

## 5. Conclusions

This study shows a significant correlation between the compositional magnetic resonance biomarkers T1ρ and T2 and age, but not for the compositional magnetic resonance biomarker T1. Our finding supports the idea that cartilage structure and composition in the metacarpophalangeal joints change during the aging process and that these changes are detectable by compositional magnetic resonance imaging. Henceforth, age-related cartilage modifications should be considered when magnetic resonance imaging studies are conducted in patients with cartilage diseases such as osteoarthritis or rheumatoid arthritis.

## Figures and Tables

**Table 1:** Detailed sequence parameters.

Sequence Parameter	T1	T1ρ	T2	Proton Density
Resolution (mm^3^)	0.5 × 0.5 × 4.5	0.5 × 0.5 × 4.5	0.5 × 0.5 × 4.5	0.3 × 0.3 × 2
Flip Angle (°)	180	15	15	150
Field of View (mm^3^)	140 × 140 × 4.5	140 × 140 × 4.5	140 × 140 × 4.5	140 × 140 × 44
Number of slices	1	1	1	20
Distance between slices (mm)	0	0	0	0.2
TE (ms)	12	2.59	2.59	39.00
T2prep (ms)	-	-	0, 10, 20, 30, 40, 50, 60, 70, 80, 90	-
TR (ms)	5190	5000	5000	3160
TI (ms)	25, 100, 500, 1000, 2000	-	-	-
Averages	1	1	1	1
Duration (min:sec)	1:50 × 5	1:12	1:12	2:51
Grappa factor	2	-	-	-
TSL (ms)	-	0, 10, 20, 30, 40, 50, 60, 70, 80, 90	-	-

Abbreviations: TE—echo time; TR—repetition time; TI—inversion recovery time; GRAPPA—Generalized Autocalibrating Partially Parallel Acquisitions; TSL—time of spin-lock.

**Table 2:** Descriptive statistics of relaxation times T1, T1ρ, and T2.

Relaxation Time	Mean	Std	Median	Min	Max
T1 (ms)	930.3	69.8	918.8	766.7	1090.2
T1ρ (ms)	20.0	4.1	19.6	15.0	32.6
T2 (ms)	15.7	3.0	15.6	9.3	25.7

**Table 3:** Percentage increase in T1ρ and T2 relaxation times with age.

Age 1 (Years)	Age 2 (Years)	Percentage Increase in T1ρ between Age 1 and Age 2 (%)	Percentage Increase in T2 between Age 1 and Age 2 (%)
20	30	6.4	4.0
20	40	12.8	8.1
20	50	19.2	12.1
20	60	25.6	16.1
30	40	6.0	3.9
30	50	12.0	7.8
30	60	18.1	11.6
40	50	5.7	3.7
40	60	11.4	7.5
50	60	5.4	3.6

## Data Availability

Data can be provided by the authors upon reasonable request.
